# Clotrimazole-Loaded Borneol-Based In Situ Forming Gel as Oral Sprays for Oropharyngeal Candidiasis Therapy

**DOI:** 10.3390/gels9050412

**Published:** 2023-05-15

**Authors:** Nutdanai Lertsuphotvanit, Sarun Tuntarawongsa, Kritamorn Jitrangsri, Thawatchai Phaechamud

**Affiliations:** 1 Program of Pharmaceutical Technology, Department of Industrial Pharmacy, Faculty of Pharmacy, Silpakorn University, Nakhon Pathom 73000, Thailand; lertsuphotvanit_n@silpakorn.edu; 2 Pharmaceutical Intellectual Center “Prachote Plengwittaya”, Faculty of Pharmacy, Silpakorn University, Nakhon Pathom 73000, Thailand; tuntarawongsa_s@su.ac.th; 3 Natural Bioactive and Material for Health Promotion and Drug Delivery System Group (NBM), Faculty of Pharmacy, Silpakorn University, Nakhon Pathom 73000, Thailand; 4 Department of Chemical Engineering and Pharmaceutical Chemistry, School of Engineering and Technology, Walailak University, Nakhon Srithammarat 80160, Thailand; kritamorn.ji@mail.wu.ac.th; 5 Department of Industrial Pharmacy, Faculty of Pharmacy, Silpakorn University, Nakhon Pathom 73000, Thailand

**Keywords:** oropharyngeal candidiasis, clotrimazole, borneol, solvent removal, in situ forming, gel

## Abstract

Oral candidiasis encompasses fungal infections of the tongue and other oral mucosal sites with fungal overgrowth and its invasion of superficial oral tissues. Borneol was assessed in this research as the matrix-forming agent of clotrimazole-loaded in situ forming gel (ISG) comprising clove oil as the co-active agent and *N*-methyl pyrrolidone (NMP) as a solvent. Their physicochemical properties, including pH, density, viscosity, surface tension, contact angle, water tolerance, gel formation, and drug release/permeation, were determined. Their antimicrobial activities were tested using agar cup diffusion. The pH values of clotrimazole-loaded borneol-based ISGs were in the range of 5.59–6.61, which are close to the pH of 6.8 of saliva. Increasing the borneol content in the formulation slightly decreased the density, surface tension, water tolerance, and spray angle but increased the viscosity and gel formation. The borneol matrix formation from NMP removal promoted a significantly (*p* < 0.05) higher contact angle of the borneol-loaded ISGs on agarose gel and porcine buccal mucosa than those of all borneol-free solutions. Clotrimazole-loaded ISG containing 40% borneol demonstrated appropriate physicochemical properties and rapid gel formation at microscopic and macroscopic levels. In addition, it prolonged drug release with a maximum flux of 370 µg·cm^−2^ at 2 days. The borneol matrix generated from this ISG obsentively controlled the drug penetration through the porcine buccal membrane. Most clotrimazole amounts still remained in formulation at the donor part and then the buccal membrane and receiving medium, repectively. Therefore, the borneol matrix extended the drug release and penetration through the buccal membrane efficiently. Some accumulated clotrimazole in tissue should exhibit its potential antifugal activity against microbes invading the host tissue. The other predominant drug release into the saliva of the oral cavity should influence the pathogen of oropharyngeal candidiasis. Clotrimazole-loaded ISG demonstrated efficacious inhibition of growth against *S. aureus*, *E. coli*, *C. albicans*, *C. krusei*, *C. Lusitaniae*, and *C. tropicalis*. Consequently, the clotrimazole-loaded ISG exhibited great potential as a drug delivery system for oropharyngeal candidiasis treatment by localized spraying.

## 1. Introduction

Oral cavity disease such as oropharyngeal candidiasis is an opportunistic infection with the fungus *Candida albicans*. It causes painful red or white lesions of the oropharynx, which can affect taste, speech, and eating. The reports on oral cavity infections by *Candida* species have increased because of the escalation in HIV infection and the acquired immune deficiency syndrome (AIDS) epidemic [1,2]. Oropharyngeal candidiasis exerts influence on 15–60% of people with hematological or oncological malignancies during periods of immunosuppression; meanwhile, it occurs in 7–48% of people with HIV infection and in over 90% of those with advanced disease [3]. Moreover, oropharyngeal candidiasis can be caused by other species such as *C. glabrata*, *C. tropicalis*, and *C. krusei* [4]. This disease can impair speech, nutritional intake, and quality of life [5].

**Figure 1:**
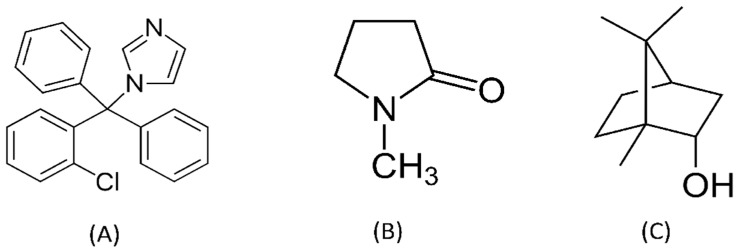
Chemical structures of clotrimazole (**A**), NMP (**B**), and borneol (**C**).

Conventionally, topical antifungals are the preferred treatment for oral candidiasis. The locally employed antifungals offer the advantage of reducing systemic exposure, which results in fewer adverse drug reactions or interactions [6]. The most conventional and efficient drugs are polyenes and azoles groups, including clotrimazole [7]. Thus, clotrimazole is an interesting choice suitable to be used as an antifungal model drug in this study. Clotrimazole exhibits lipophilic property, and it is freely soluble in alcohol and soluble in polyethylene glycol 400, acetone, and chloroform [8]. Moreover, it exhibits a poor aqueous solubility (0.49 μg/mL) [9] that may affect its antimycotic activity. The structure of clotrimazole is shown in Figure 1A. Clotrimazole is a wide-spectrum triazole-based antifungal agent. It inhibits the enzyme cytochrome P450 14α-demethylase, which is required in fungal cell membrane synthesis, thereby affecting the permeability of the cell membrane. This generates the leakage and loss of essential intracellular compounds and eventually causes cell lysis [10]. It has antimycotic activity against *Candida* spp., *Cryptococcus* spp., dermatophytes, and *Aspergillus* spp. [11]. In addition, it displays activity against certain Gram-positive bacteria such as *Staphylococcus aureus* and *Streptococcus pyogenes* [10,12]. The drug is commonly used for skin, oral, and vaginal candida infections, typically given topically or as oral or vaginal troches, and has only limited systemic absorption [12]. Moreover, topical forms of clotrimazole are available as over-the-counter medication and are considered reasonably safe and without serious side effects. The troches are FDA-approved for children 3 years of age and older [13].

The efficiency of clove oil has been described for its antibacterial activities against various cariogenic and periodonto-pathogenic bacteria [14]. It has been generally regarded as safe (GRAS) and exerts an antimicrobial activity against many Gram-positive and Gram-negative organisms as well as some fungi, with anesthetic properties [15,16]. The constituents of clove oil, such as eugenol, oleic acids, and lipids, contribute to its antimicrobial activity [17]. This oil is also applied in dental cement, filler, and restorative material [14]. In higher amounts, it promotes the antimicrobial activity of zinc oxide gel [18]. The hydrophobic nature of essential oil is attributed to the greater permeability of the bacterial cell membrane where the extensive leakage of bacterial cells results in cell death [19]. Therefore, the combination of clove oil and clotrimazole is interesting as the active components of a dosage form for the treatment of oral candidiasis. In this study, the combination between clotrimazole and clove oil was expected to be formulated as an oral antifungal spray for topically covering a lesion area of the oral cavity.

In oropharyngeal candidiasis therapy, topical treatments with local administration are becoming increasingly popular because of greater patient compliance and low side effects. Topical dosage forms for treatment of oral candidiasis are administered via suspensions, mouth rinses, gels, and troches. However, salivary clearance can rapidly diminish the drug level in the affected area. Usual administration is via 10 mg lozenges (troches), which dissolve slowly in the mouth, five times daily for 7 to 14 days [13]. An ideal dosage form for the treatment of oral candidiasis should provide sustained drug release and produce an antifungal effect for a prolonged period. Thus, the in situ forming gel (ISG) system is interesting for application in this work to prolong the drug release and overcome this problem. The major challenges of the local drug delivery system in the oral cavity are in designing a formulation with the ability to adhere to the target organ, assist with a prolonging drug release, and still exhibit antimicrobial activities. A solvent-removal-induced ISG system is one of the satisfactory approaches for controlled drug delivery. Its liquid state can be transformed to the solid state by phase inversion via the solvent-removal mechanism [20,21,22]. This ISG consists of a drug and a water-insoluble matrix-forming agent dissolved in a biocompatible, water-miscible organic solvent such as *N*-methyl-2-pyrolidone (NMP) (Figure 1B). When this solution contacts an aqueous environment such as saliva, the organic solvent diffuses outward into the surrounding environment, while water diffuses into the system, inducing phase inversion of the matrix-forming agent to form a gel state before completely forming into a solid matrix and entrapping the drug for slow release over time [22,23].

The key substance in a solvent-removal-induced ISG system is the matrix-forming agent, which should be a non-toxic and water-insoluble matter that is soluble in a water-miscible organic solvent such as NMP. Borneol (Figure 1C) is a bicyclic monoterpene that has been used in food flavoring, air care products, decorative cosmetics, traditional Chinese medicine, etc. [24]. It relieves local itching, bronchitis, and minor aches and pains of muscles and joints [25]. In addition, it has an antibacterial effect and antinociceptive effect; in addition, it has been developed as a drug delivery system [23]. It is slightly denser than water, insoluble in water, soluble in alcohol, and slightly soluble in propylene glycol [26]. The low toxicity of borneol has been reported for skin irritation on human volunteers and mice/mouse [24]. Borneol significantly reduced the nociceptive behavior at the early and late phases of mice paw licking and reduced the writhing reflex and the carrageenan-induced leukocyte migration to the peritoneal cavity without impairing motor coordination in mice [27]. It was considered as a safe and effective penetration enhancer for ocular drug administration because it improved the transcorneal penetration of both hydrophilic and lipophilic compounds without causing toxic reactions [28]. The preparation of vancomycin HCl-loaded borneol-based in situ forming matrices have been reported for periodontitis treatment [23]. They prolonged drug release and efficiently inhibited many microbes, including methicillin-resistant *S. aureus* (MRSA). Because of its safe long-term use, apparent low aqueous solubility, inexpensiveness, and advantageous nature, borneol served as the potential matrix-forming agent of the clotrimazole-loaded ISG system in this research.

The aim of this study is to develop a clotrimazole-loaded ISG formulation as an oral antifungal spray for covering a lesion area of the oral cavity by using borneol as the matrix-forming agent for oropharyngeal candidiasis treatments. To achieve the formulation development, understanding matrix-forming behavior of borneol is crucial. Thus, the influence of different concentrations of borneol was studied and discussed in terms of physicochemical properties, matrix-forming behavior, and the effectiveness of ISG. The assessment of prepared clotrimazole-loaded borneol-based ISG formulations was investigated via the issue of density, pH, viscosity, rheology behavior, surface tension, contact angle, spray pattern, matrix-formation behavior, drug release and permeation, and antimicrobial activities.

## 2. Results and Discussion

### 2.1. Physical Appearance

The successful preparation of ISG solutions was attained with different concentrations of borneol (20–50% *w*/*w*) individually combined with 1% *w*/*w* clove oil and 1% *w*/*w* clotrimazole dissolved in NMP as the formula presented in the Materials and Methods section. The homogeneous and clear solutions of CN (clove oil in NMP), ZN (clotrimazole in NMP), ZCN (clotrimazole and clove oil in NMP), and 40BN (40% *w*/*w* borneol in NMP) were also obtained as the comparative groups. Clotrimazole and clove oil were dissolved in NMP over a short time during preparation by simple mixing because they could freely solubilize in this solvent. The borneol concentration at more than 50% *w*/*w* dissolved in NMP with difficulty, and some precipitates of excess borneol powders appeared. The successful formulation of clotrimazole-loaded borneol-based ISGs was indicated by transparent, colorless, and low-viscosity solutions. The incorporation of clotrimazole or increasing the borneol content in ISG solutions did not remarkably change the visual appearance. The addition of clove oil to borneol-based ISGs only turned the colorless solution to a clear yellowish solution because clove oil has a yellowish color.

### 2.2. pH and Density

The pH of the preparations was checked to define their irritation of the contact tissue. The pH values of the solvent, drug solutions, and ISGs are shown in Table 1. The pH values of NMP and CN were significantly higher than those of the other formulations (*p* < 0.05). The pH decreased after adding clotrimazole or clove oil and apparently decreased gradually after subsequently increasing the borneol content because of the smaller amount of NMP in formulation [29]. The pH values of drug-loaded borneol-based ISGs were in the range of 5.59–6.61; therefore, they were acceptable because these values are close to the pH of 6.8 of saliva in the oral cavity. Additionally, a spray amount of dosage form around 0.5 mL is normally applied during administration to the oral cavity. Thus, the rather high flow rate of saliva could dilute the acidity from ISG and maintain its local environmental pH of 6.8 [30].

The density value of NMP was 1.03 ± 0.01 g/cm^3^ and nearly constant after adding clove oil and clotrimazole (Table 1). There was no statistical difference for density value between those of NMP and the others. A slight decrease in density was found with more addition of borneol in the formulation because the density of borneol is 1.01 g/cm^3^ [31]. These ISGs were slightly heavier than water; therefore, they should not float after spraying into an aqueous medium and submerge while the matrix is formed at the targeting site. Additionally, these presented density values were further applied as the necessary parameter for consequently determining surface tension.

### 2.3. Surface Tension

From drop-shape analysis, the fluid suspended in air with the cohesive force of liquid molecules contracts to the smallest possible droplet surface area [32]. Therefore, the interference of this cohesive force with the additional incorporated excipient that enables the formation of an adhesive force could influence the surface tension of the mixture fluid [33,34]. In addition, the lower surface tension promotes the spreadability of formulations on the tissue surface because of the decreased cohesive force of the fluid droplets. The obtained values of the surface tension of formulations are displayed in Table 1. The addition of clotrimazole increased the surface tension compared to the clove oil, whereas greater additions of borneol gradually decreased this value. The surface tension of borneol-based ISG solutions was significantly (*p* < 0.05) less than the non-loading borneol formulations. This result indicates that clove oil and clotrimazole could interact with the NMP molecule and be submerged in the bulk fluid phase instead of interacting on the droplet surface. The diminishment of surface tension due to more borneol addition demonstrated that the cohesive force among NMP molecules around the surface region was disturbed by adhesion force between NMP-borneol molecules and they emerged on droplet surface. The polar aprotic solvent, i.e., NMP, has stronger hydrogen bonding interactions between molecules than the borneol–borneol or borneol–NMP molecules [33,34]. Therefore, the tendency of decreased surface tension owing to the borneol addition could provide an advantage of ease of spraying and spreadability of the formulation for delivery to the target site area, such as the oral cavity membrane, for treatment of oropharyngeal candidiasis.

### 2.4. Viscosity and Rheological Behavior

**Figure 2:**
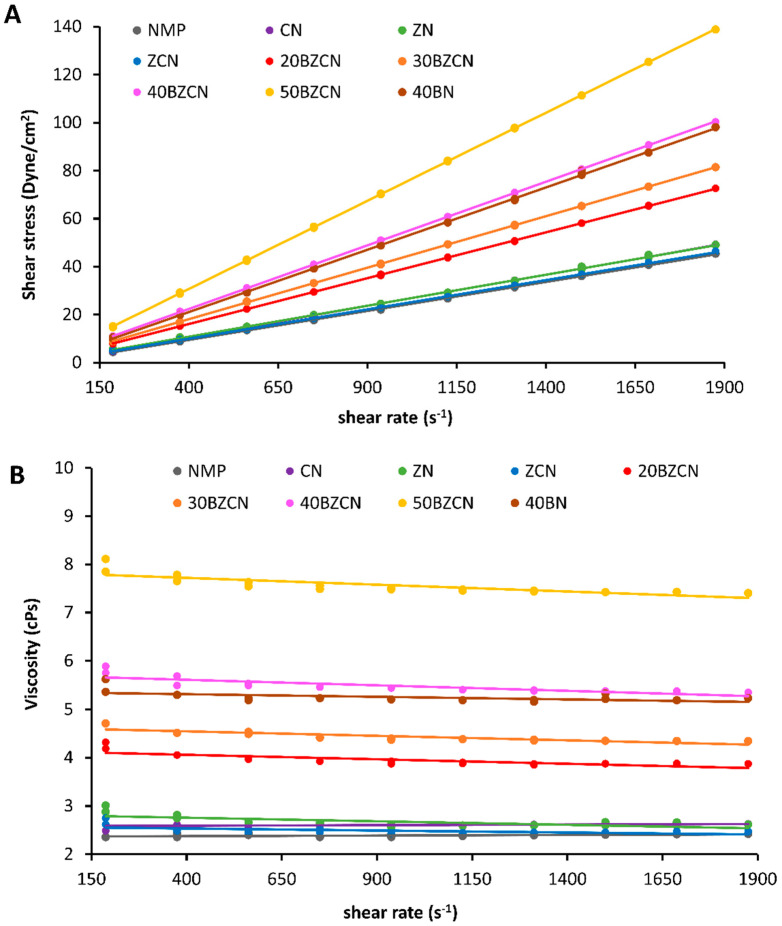
Rheological behavior of clotrimazole-loaded borneol-based ISG and related formulations, plotting between shear stress against shear rate (**A**) and relationship between viscosity and shear rate at room temperature (**B**).

The viscosity of borneol-based ISGs was significantly (*p* < 0.05) higher than non-loading borneol formulations, as presented in Table 1 and Figure 2A. The addition of clove oil, clotrimazole, and more borneol increased the viscosity of the systems because the decrease in solvent content led to an increased solutes concentration and environmental viscosity. Nonetheless, all of them exhibited a prominently low viscosity (<8 cPs) compared with typical polymer-based ISG because of the low density, small size, and unsophisticated structure of borneol loaded in NMP [35,36,37,38]. These obtained viscosity data also corresponded with the previous report that the viscosity of vancomycin HCl-loaded borneol-based ISG using DMSO as a solvent for periodontal pocket delivery was noticeably lower than 20 cPs [23]. NMP is claimed as an important vehicle in pharmaceuticals, utilized for drug extraction and crystallization, and mentioned to enhance the solubility and permeability of drugs by simultaneously acting as a co-solvent and a complexing agent. The strong hydrophilic interaction between the hydroxyl group of borneol and the keto group of NMP decreased NMP molecular movement and increased the viscosity [39]. Notably, the viscosities of the clotrimazole-loaded borneol-based ISGs were outstandingly lower than previously mentioned on polymer-based in situ forming systems [35,36,37]. In addition, these developed ISGs also showed lower viscosities than natural resin-based ISGs [40,41,42]. These natural resins, including rosin, benzoin, propolis, and aloe, dissolved in DMSO and NMP were employed as the gelling agents of ISGs in which the viscosities of benzoin in NMP and DMSO were 6.61–171.46 and 19.77–137.03 cPs, respectively, compared with propolis in NMP and DMSO (i.e., 119–155 and 80.96–577.90 cPs, respectively) [41]. The rather low viscosities (<20 cPs) of ibuprofen-based ISGs were recently reported using NMP and DMSO as the solvents [43]. The advantage of the markedly low viscosity of these clotrimazole-loaded borneol-based ISGs was gained, including their simplicity of mixing for fabrication and comfortability for spraying. The beneficial characteristics were also reported for ISGs fabricated using lauric acid as a matrix-forming agent [44,45,46]. Moreover, they showed the Newtonian flow as displayed in Figure 2B, which is mentioned as one of the appropriate flow behaviors for spraying through a spray nozzle to the oral cavity [47].

### 2.5. Contact Angle

**Figure 3:**
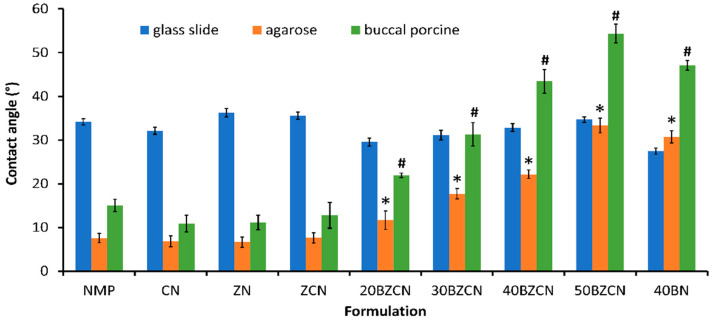
Wettability of clotrimazole-loaded borneol-based ISG and related formulations on the surface of the glass slide, agarose gel, and buccal porcine. Results are shown as mean values ± SD and demonstrated at room temperature (*n* = 3). * and # represent a significant difference (*p* < 0.05) compared with ZCN.

Normally, the measurement of the contact angle could be conducted using a goniometer on a standard glass slide. This study was also designed to test the contact angle of ISGs on agarose gel and the porcine buccal membrane. This test aimed to determine the wettability or spreadability of the ISG on the target surface. On a glass surface, a similar and rather high contact angle value in the range of 33–38 degrees was evident for borneol-free solutions (Figure 3). The incorporation of borneol decreased this value on the glass surface, indicating the increased hydrophobicity of the formula and enhanced adhesive manner; nonetheless, the gradual increase in viscosity resulted in an enhanced contact angle owing to more difficulty in fluid spreadability [43,48]. In addition, the more highly viscous 40BZCN had a greater contact angle value than 40BN (Figure 3). The rather high contact angle value of 30% and 40% of the ibuprofen-based ISG was attributed to its high viscosity, retarding the spreadability of the formulations on the glass surface, as also previously reported [43]. Senarat et al. also formerly reported the higher contact angle value of rosin-based ISG on a glass slide [48].

To mimic the environment of buccal mucosa covered with saliva, this study used the surface of an agarose gel containing phosphate ion species and water as another target surface. Owing to its smooth planar appearance, agarose gel proved a suitable surface for checking the spreadability of ISG [43]. Remarkably, the contact angle of borneol-loaded ISGs was significantly (*p* < 0.05) higher than that of the borneol-free formula, such as that with ZCN. The solvent-removal-inducing phase inversion after contact with water from the agarose gel changed the solubilized borneol phase to a matrix-like one and decreased the spreadability. The increased amount of borneol loading resulted in a greater contact angle on the surface of the agarose gel; meanwhile, the good miscibility of NMP and water showed to apparently lower the contact angle of the borneol-free formulations (Figure 3).

Similar results were found on the surface of the porcine buccal mucosa, even though there were particularly higher contact angle values than for the surface of the agarose gel. Moreover, they also exhibited a significantly (*p* < 0.05) and noticeably higher contact angle than the borneol-free formulations. The direct contact of borneol-loaded ISGs with the buccal mucosa soaked with phosphate buffer saline solution (PBS) provoked more rapid solvent removal from the formulation than on agarose gel. The more highly viscous fluid of 40BZCN retarded solvent removal and showed a slightly lesser contact angle value than that of 40BN [49]. This consequence could be attributed to the fact that the incorporation of other solutes could minimize the cohesive force between NMP molecules, as previously presented in the surface tension results; thereafter, the extent of spreading increased, whereas the contact angle decreased, indicating the difficulty in matrix formation. This evidence corresponded with the ibuprofen-based ISGs in DMSO and NMP, which increased the ibuprofen loading and decreased the contact angle on agarose gel [43]. Even though there were rather higher contact angle values for the developed ISGs, they were apparently less than 60°, manifesting their good wettability on these test surfaces [40,43]. Typically, the wettability of ISGs is required for their adhesiveness onto buccal mucosa to prevent the undesirable dissipation of the transformed clotrimazole-loaded borneol matrix [50].

### 2.6. Microscopic Changes of In Vitro Gel Formation

**Figure 4:**
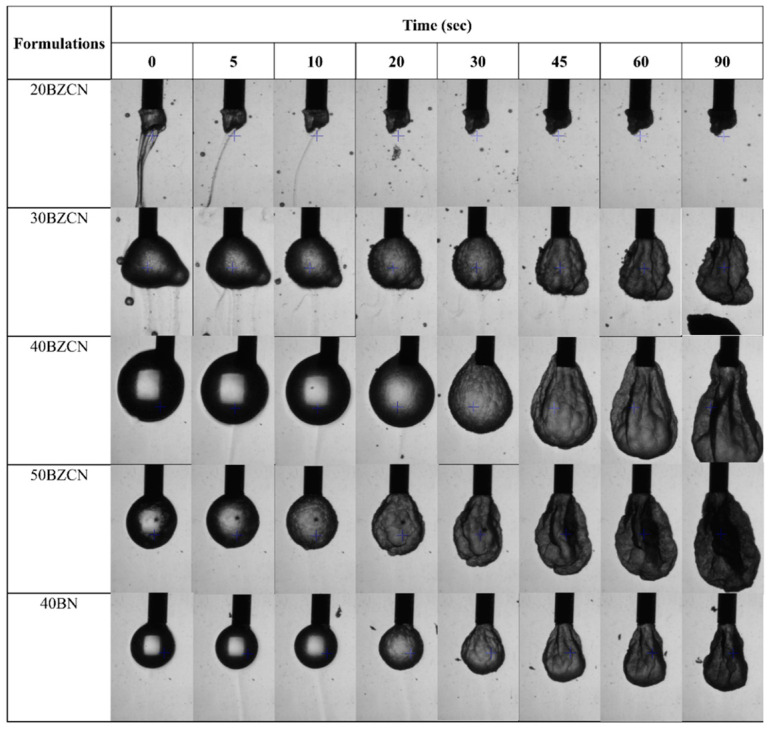
Interfacial tension and gel-formation behavior of clotrimazole-loaded borneol-based ISGs and borneol solution in PBS with pH 6.8.

The morphological changes of the drug-loaded ISGs after pulling out and contact with PBS with pH 6.8 as the surrounding medium are shown in Figure 4. The 20BZCN liquid flowed with gravitational force to the bottom of the medium because it did not transform sufficiently into matrix droplets. This low concentration of borneol in the ISG enabled a relatively inadequate phase transformation because of the low affinity of its formulation in the water environment and the small amount of matrix-forming agent [44]. The 30BZCN formed an irregularly shaped droplet, with a huge amount of its liquid formulation flowing downward initially, and then, the size gradually decreased. Further shrinkage of the matrix droplet was noticeable, and it remained suspended at the needle tip. This behavior of matrix formation was found for 40BZCN, with less leakage of the liquid from the initial droplet. In addition, the spherical droplet was generated and remained from the initial time to 20 s and then shrunk into an obovate matrix with a rough surface. This similar mode of transformation occurred for 50BZCN; however, its demonstrated a smaller spherical shape, and slight leakage of the liquid occurred. Therefore, increasing the amount of borneol in the ISG hastened the transformation into a hard matrix with smaller and more spherical-shaped droplets before subsequent shrinkage into a smaller, obovate, and rough matrix after their solvent removal. The increase in borneol mass could retain the inner liquid droplet, which did not diffuse out. The leakage of the liquid from the 40BN droplet was similar to that of 40BZCN; nevertheless, the size of the obtained droplet of 40BN was particularly smaller. This shrinkage of droplets also indicated the solvent removal from ISG during its phase transformation; therefore, the initial spherical-shaped droplet could not retain its shape after more NMP diffused outward into PBS owing to this solvent’s high affinity and good miscibility to water. There were no floating droplets, as found in the case of lauric-acid-based ISG [44], because of the higher density of borneol. The present investigation provides not only an insightful new experiment for tracking ISG systems but also a fruitful assessment tool for evaluating the self-forming behavior of in situ forming dosage forms at the microscopic scale.

### 2.7. Macroscopic Changes of In Vitro Gel Formation

**Figure 5:**
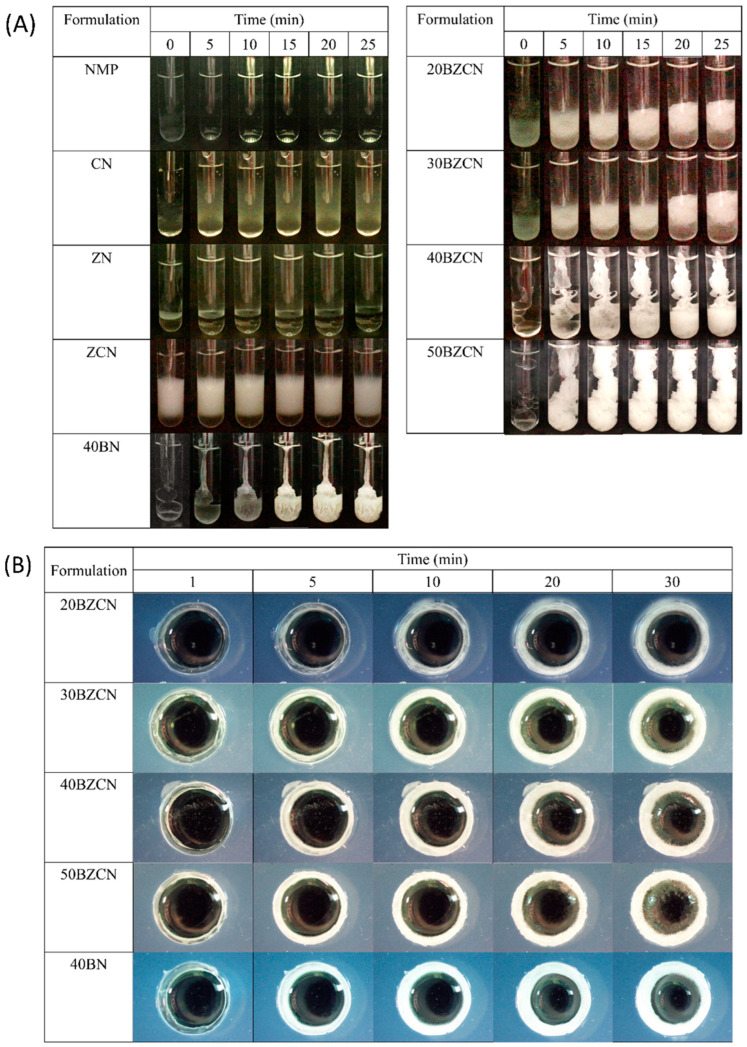
(**A**) Change of gel formation with time after contact with PBS with pH 6.8. (**B**) Morphological change of clotrimazole-loaded borneol-based ISG and related formulations after exposure with agarose gel containing PBS with pH 6.8.

The photographs taken after all prepared systems’ exposure to PBS with pH 6.8 are shown in Figure 5A. NMP and ZN only sank to the bottom of test tube owing to their higher densities, whereas CN and ZCN showed a colloid-like response in PBS because clove oil was dispersed in the aqueous medium. The increased insoluble compounds in ZCN, including clotrimazole and clove oil, promoted cloudier liquid. A cloudy matrix gradually presented for 40BN. The drug-loaded ISGs comprising borneol less than 40% *w*/*w* showed as a loose, cloudy mass that expanded itself upward, while the lower part remained in liquid form. This result indicated that the low-concentrated borneol-based ISG did not form properly into an interconnected, dense matrix. The 50BZCN presented a more rapid phase transformation into a dense, opaque matrix compared to 40BZCN. The abrupt matrix formation of 50BZCN was evident, whereas a step-by-step change from solution to gel state and cloudier matrix was obtained for 40BZCN. This incidence was also observed for other ISGs comprising different concentrations of polymers [29,35,36,37], natural resins [40,41,42,48], and fatty acids [44,45,46]. In addition, the appropriate concentration of matrix-forming agents from these previous reports reached nearly 40% *w*/*w* because of their fast, dense matrix formation that was, however, not too rapid before formulation spreading at the target site. The sudden change from solution to matrix of 50BZCN might hamper sufficient spreading on the buccal mucosa. By comparison, the 40BZCN matrix had a greater mass compared to that of 40BN because the drug-loaded formulation was composed of more aqueous-insoluble ingredients.

A cross-sectional view of the phase transformation of drug-loaded borneol-based ISGs in an agarose well is depicted in Figure 5B. The 20BZCN showed as a rather transparent gel that occurred surrounding the agarose rim and gradually became cloudier, signifying its rather low-density matrix formation owing to the lowered amount of borneol. A thicker matrix formation was noticeable for 30BZCN compared to the others. Because the higher concentration of borneol created a denser matrix as a barrier for solvent removal, the phase transformation was retarded. This characteristic result has been previously mentioned in the case of fatty-based ISGs [44,45,46]. Theoretically, the thermodynamic stability could be interrupted by various factors such as the presence of new interfacial energy from the initial crystal, aqueous surface, bubble surface, etc., together with the supersaturation from continuous solvent removal such as NMP leakage. Thereafter, the ISG stabilized itself, leading to further matrix formation [51]. A larger cloudy mass in the liquid phase of the 50BZCN formulation was a result of a later matrix. The trend of a diminishing transformation rate by time shown by 40BZCN and 50BZCN was caused by the restriction of solvent transfusion through the interfacial network of the initiated borneol matrix, which became denser with lower porosity. Similarly, the dense gelation network also lowered the water diffusion coefficient [52]. These microscopic and macroscopic matrix formations were beneficial in demonstrating the performance of 40BZCN in its gradual phase transformation to a matrix, with a sufficient amount of matrix mass for spreading on the target area.

### 2.8. Spray Pattern

The spray angle and pattern of test solutions were determined. A wider angle was found for borneol-free solutions. The NMP showed the widest spray angle (61.70 ± 0.94°) and scatter than the other solutions. The solution containing both clotrimazole and clove oil (ZCN) exhibited a narrower spray angle (48.60 ± 1.36°) than that containing clove oil (CN) (52.83 ± 0.81°) and clotrimazole (ZN) (56.20 ± 2.44°), respectively. The 40BN had a spray angle of 41.81 ± 1.43°. By comparison, an addition of borneol, e.g., 20BZCN, created a wider spray angle (51.94 ± 1.19°) than ZCN. This result could be attributed to the lubricating effect of borneol; nevertheless, the enhanced viscosity from the higher concentration of borneol minimized the spray angle and exhibited lesser droplet scatter. The spray angles of 30BZCN, 40BZCN, and 50BZCN were 48.96 ± 0.69°, 46.72 ± 1.05°, and 40.46 ± 0.72°, respectively. Therefore, there was a progressive decrease in spray angle as the concentration of borneol increased. Notwithstanding, the spray angle and pattern of 40BZCN could be acceptable for application in the oral cavity.

### 2.9. Water Tolerance

**Figure 6:**
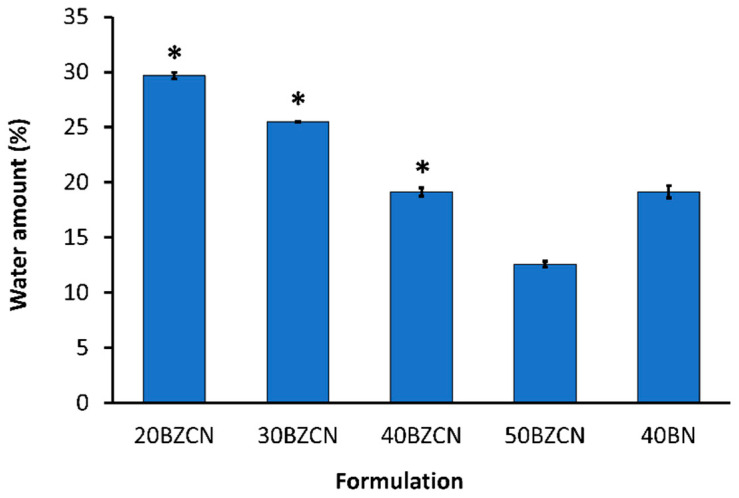
Percentage of water inducing a phase separation of clotrimazole-loaded borneol-based ISGs and borneol solution. Results are shown as mean values ± SD, (*n* = 3). * represents a significant difference (*p* < 0.05) compared with 50BZCN.

The percentage of water that induced phase separation of the developed clotrimazole-loaded borneol-based ISG solutions from a clear solution to turbid fluid by water titration is presented in Figure 6. These obtained data informed the water sensitivity of formulations or their ease of phase inversion due to the enhanced polarity of the dispersed medium. Therefore, the minute value of the percentage of water- inducing a phase separation signified that formulation’s lesser water tolerance [43,44]. The decrement of the % water that induced phase separation conceivably occurred as the amount of borneol increased (Figure 6). This 50BZCB value was significantly (*p* < 0.05) less than the other formulations. The greater the incorporated dissolved hydrophobic compound, such as borneol, the more sensitive the formulation against the aqueous phase. The 40BZCN and 40BN showed a similar % water able to induce phase separation, even though the drug, clove oil, and borneol combined in 40BZCN diminished the amount of solvent in the system. In addition, the lipophilicity of the system was subsequently diminished when more water was introduced into the formulation because the dielectric constant of water is higher than that of NMP. The dielectric constant values of NMP and water are 32 and 78.54, respectively [53,54]. The high affinity and good miscibility between NMP and water could be explained according to hydrogen bonding and dipole–dipole interaction. Iamir et al. reported that this bond is stronger than that between water molecules and is attributed to a complex at the molal fraction ratio 2:1 of the water–NMP solution; meanwhile, an increase in temperature might tighten this interaction [54]. Their beneficial miscibility resulted in high-potential solvent exchange and promoted the diffusion out of NMP from ISG into the aqueous phase. This solvent removal of ISG promoted phase inversion and matrix formation after application to the target area, such as the buccal mucosa. A similar result was reported for antibiotic-drug-loaded ISGs prepared using ibuprofen and lauric acid as the matrix-forming agents, in which a decreasing water tolerance presented as their concentration of matrix-forming material increased [43,44]. Nevertheless, this technique for measurement of water tolerance was difficult to be applied to polymeric or natural resin-based ISGs because the added water did not diffuse homogeneously in these rather viscous formulations. Therefore, only spot phase separation occurred at the upper part of these formulations and did not attain the % water-tolerance value accurately. For the less viscous ISGs using a non-polymer or non-resin as the matrix-forming agent, the homogeneous phase separation occurred due to moderate mixing, and their percentage of water tolerance could be attained fortuitously.

### 2.10. Drug Release and Permeation

**Figure 7:**
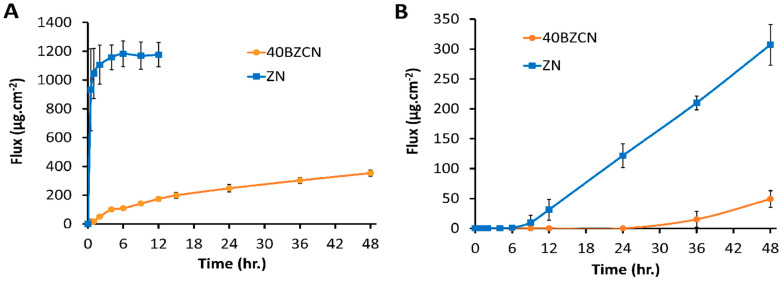
Clotrimazole release through (**A**) nylon membrane and (**B**) permeability through porcine buccal membrane from 40BZCN compared with ZN (*n* = 6).

*C. albicans*, an omnipresent commensal organism, typically colonizes on oral mucosa. The distinct morphological states of *C. albicans* dictate the phases of colonization, growth, and dissemination, where the yeast form is associated with both initial attachment and dissemination, while the hyphal form enables *C. albicans* to invade host tissue [55,56]. Besides an active penetration, another complementary mechanism of *C. albicans* for host cell invasion is endocytosis, a passive, fungal-induced, but host-cell-driven process whereby lytic enzymes and invasins expressed on hyphae bind to and degrade E-cadherin and other inter-epithelial cell junctional proteins, enabling the organism to penetrate between epithelial cells [57]. Therefore, the diffusion ability of an antifungal drug from a formulation through the oral cavity mucosa, such as the buccal membrane, should be investigated. The release behaviors of clotrimazole from ZN and 40BZCN through nylon membrane using the Franz diffusion apparatus are shown in Figure 7A. Clotrimazole rapidly diffused from ZN through the nylon membrane and reached a plateau state at 2 h, which was ostensibly faster than 40BZCN. The maximum flux value of 40BZCN was 370 µg/cm^2^ at 2 days. The borneol matrix formation after this formulation contact with the soaked nylon membrane slowed the drug diffusion from 40BZCN. The greater tortuosity of the borneol matrix from the gradual solvent-removal process enabled retardation of the clotrimazole diffusion into the receiving medium in the receptor. It is worth mentioning that 40BZCN efficiently prolonged the release of clotrimazole. The decrease in release rate of propranolol HCl in the presence of halloysite nanotubes in the polyvinyl alcohol transdermal gel was observed due to the loading of the drug into the material lumen or the strong ion interaction between the drug and the halloysite nanotube [58]. The higher viscosity of topically used clotrimazole-loaded microemulsion was a driving force for retardation of the drug release [59]. The aforementioned result confirmed the ability of the developed removal-based ISG, e.g., with 40BZCN, for prolonging the release of clotrimazole.

The permeation behaviors of ZN and 40BZCN through the porcine buccal membrane are presented in Figure 7B. A more significant amount of clotrimazole was liberated and penetrated from ZN through the porcine buccal membrane to a receiving medium in the receptor compared to the performance of 40BZCN. In addition, the lag time of drug release of ZN was shorter than that of 40BZCN, which was 7 h and 26 h, respectively. Therefore, the buccal membrane performed as a barrier for drug penetration, and the borneol matrix generated from 40BZCN ostensibly controlled the drug penetration. This drug’s permeation through the porcine buccal membrane was undertaken in parallel with drug release through the nylon membrane for 48 h. The clotrimazole’s release and permeation behaviors could be compared between selected test formulations from the duration period of these studies. The saliva flow and meal intake could limit its adhesion onto the oral mucosa; nevertheless, the prolongation ability for drug release and permeation of the developed ISG should be useful for efficient treatment of oropharyngeal candidiasis. In addition, the ability of drug permeation could promote the antimicrobial activities for invader microbes in the mucosa tissue. Biocompatible and biodegradable spray is the most appropriate dosage form for buccal application due to its versatility, adaptability, physical flexibility, comfort, lightness, acceptability, and adjustable size; therefore, it enhances patient compliance compared to bio-adhesive tablets [60]. The accumulations of clotrimazole in the donor buccal membrane and the receiving medium are shown in Table 2. There was no residue remaining for analyzing the drug content in a donor or in the formulation of ZN, indicating this solution’s notable ability for drug penetration. NMP has been claimed as a skin penetration enhancer due to it increasing lipid fluidity and has presented a substantial increase in the penetration of various drugs [61]. It could support the penetration of clotrimazole; therefore, a significantly higher amount (*p* < 0.01) of drug retention was found in both the buccal mucosa and ISG compared to the receiving medium. However, this hydrophobic drug mainly accumulated significantly (*p* < 0.01) more in tissue than in the aqueous medium. For 40BZCN, the drug amount still remained more significantly in the formulation at the donor part than in the buccal membrane and receiving medium, respectively (*p* < 0.01) (Table 2). This result corresponded with the previous result of the drug release exhibiting the ability of the borneol matrix for retardation of drug diffusion and penetration through the buccal membrane. Nevertheless, some accumulated clotrimazole in the buccal mucosa showed its potential antifungal activity against invader microbes in host tissue such as the buccal area. Normally, oral candidiasis encompasses fungal infections of the tongue and other oral mucosal sites, with fungal overgrowth and its invasion of superficial oral tissues [4,7]. Thus, some permeations of clotrimazole are beneficial for antifungal activity against invader microbes. The slight amount of the drug in the receiving medium signified a lesser amount of clotrimazole penetrating into the blood stream, which did not satisfy the aim of this developed ISG. This clotrimazole-loaded borneol-ISG was designed as a localized drug delivery system for the treatment of oropharyngeal candidiasis in the oral cavity. Thus, the other predominant drug release from the 40BZCN formulation into the saliva of the oral cavity could better influence the pathogen of oropharyngeal candidiasis.

### 2.11. Antimicrobial Activities

**Figure 8:**
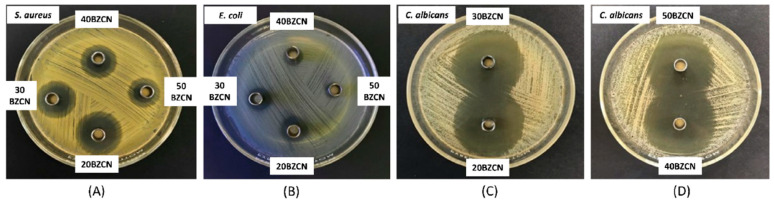
Photographs of the inhibition zone of ISG formulations containing 20–50% borneol (20BZCN-50BZCN) against *S. aureus* (**A**), *E. coli* (**B**), and *C. albicans* (**C**,**D**) (*n* = 3).

The diameters of the inhibition zone of the developed formula against *S. aureus*, *E. coli*, *C. albicans*, *C. krusei*, *C. lusitaniae*, and *C. tropicalis* from the antimicrobial activity tests via the cup agar diffusion method are presented Table 3. The photographs of inhibition zone of the developed formula against *S. aureus*, *E. coli* and *C. albicans* are presented in Figure 8. *S. aureus* and *E. coli* were employed as standard Gram-positive and Gram-negative bacteria, respectively. All tested candida species were selected because all of them are associated with oropharyngeal candidiasis. NMP is a colorless dipolar aprotic solvent that is miscible with water as well as with the most commonly used organic solvents. This is because of its nonvolatility and very good performance for dissolving diverse materials; therefore, it is used as a component in some pharmaceutical dosage forms because of its safety with low toxicity (LD_50_ of 4.15 g/kg) [22,54,62]. Furthermore, NMP has been utilized as a co-solvent for increasing the equilibrium solubility of several drugs and drug-like compounds [62]. Commercial products for periodontitis treatment such as Atridox^®^ are composed of 33.3% *w*/*w* NMP to dissolve the polymer and doxycycline hyclate [63]. Therefore, NMP was employed as a solvent for these developed ISG formulations. As shown in Table 3, NMP exhibited notable antimicrobial activities against all test microbes, especially *C. albicans*, *C. lusitaniae*, and *C. tropicalis*. NMP’s efficient inhibition against *C. candida* and bacteria growth has been formerly reported [43,64]; thus, it might promote the antimicrobial activities of developed ISGs. The addition of clove oil actually increased antimicrobial activities against all test microbes except *C. krusei*. Clove oil has been reported for its good antibacterial effect, ability to heal wounds faster, and its ability to diminish the microbial load in wounds [65]. Clove oil causes a considerable reduction in the quantity of ergosterol, a specific fungal cell membrane component [66]. Moreover, the germ tube formation by *C. albicans* was almost completely inhibited by clove oil. Therefore, clove oil has considerable antifungal activity against clinically relevant fungi, including fluconazole-resistant strains [67]. Lauric-acid-based ISGs comprise a higher amount of clove oil, contributing to a larger inhibition zone especially against *C. albicans*, which has been reported previously [68,69].

Although clotrimazole is an antifungal drug, ZN increased the inhibition efficacy of NMP, whereas it slightly affected *E. coli* (Table 3). The remarkable increase in the clear zone against *S. aureus* was evident because clotrimazole displays activity against certain Gram-positive bacteria such as *S. aureus* and *S. pyogene* [10,12]. The ZN markedly inhibited all test *Candida* species whose clear zones were larger than 36 mm. The addition of clove oil and clotrimazole in NMP (ZCN) slightly changed the inhibition clear zone from the ZN formulation. Borneol addition at 20% *w*/*w* (20BZCN) increased the clear zone of the formulation especially against *Candida* species but not against *E. coli*. Antifungal activity of borneol has been reported against planktonic *C. albicans* cells as well as its inhibition on preformed biofilms [70]. Vancomycin HCl-incorporated borneol-based in situ forming matrices have been reported for their proficiently inhibited various microbes and designed for periodontitis treatment [23]. Loading borneol higher than 20% *w*/*w* tended to gradually decrease the inhibition zone against all test microbes. The inhibition clear diameter of 20BZCN against *C. albicans* was significantly (*p* < 0.05) larger than that of 30BZCN, 40BZCN, and 50BZCN, respectively. In addition, their values are significantly different each other, as shown in Table 3. Meanwhile, the inhibition clear diameter of 20BZCN against *C. krusei* was significantly (*p* < 0.05) larger than those of 30BZCN and ZN. The inhibition clear diameter of 20BZCN against *C. lusitaniae* was significantly (*p* < 0.05) larger than those of 30BZCN, ZN, and ZCN. The inhibition clear diameter of 20BZCN against *C. tropicalis* was significantly (*p* < 0.05) larger than that of 30BZCN. Therefore, the borneol matrix occurred after phase transformation via solvent removal and retarded the diffusion of antimicrobial agents and NMP from borneol-based ISGs. These results resembled other ISGs fabricated using rosin [40,41], ethyl cellulose [29], and some fatty acids [44,45,46] as matrix-forming materials. An increased concentration of these matrix-forming materials diminished the inhibition zone against pathogens of periodontitis due to their ability to retard drug diffusion into inoculated agar media. The increase in borneol concentration could promote a greater formation of matrix mass as a barrier to retard the diffusion of all dissolved compounds and solvents; thereafter, a smaller inhibition zone can be attained. Nonetheless, the efficient inhibition against *Candida* species with a wide-diameter clear zone was achieved. As promised, the localized clotrimazole-loaded borneol-based ISG should therefore present a competent suppression of the pathogens and related microbes of oropharyngeal candidiasis. The MIC values of clotrimazole against *S. aureus*, *E. coli*, *C. albicans*, *C. krusei*, *C. lusitaniae*, and C. *tropicalis* are 31.25, >32, 0.008, 0.125, 0.008, and 0.008 µg/mL, respectively [71,72,73]. Clotrimazole is a dominant antifungal agent with a slight antibacterial effect; thus, the MIC value against *S. aureus* and *E. coli* is rather higher than against *Candida* spp. In general, the volume from one puff of an oral medication spray is around 50–200 µg. Assuming 100 mcg per puff and two consecutive puffs per dosing, an ISM of 200 µg is sprayed into oral cavity, i.e., equivalent to 1.542 µg of clotrimazole in formulation (1% *w*/*w*) when calculated from the clotrimazole remaining on the buccal mucosa of porcine tissue (77.10%), as aforementioned in Table 3. Normally, the volume of saliva in the mouth of adults is around 0.52–2.14 mL, while its average is 1.07 mL [74]. Therefore, the concentration of clotrimazole from ISM sprayed into the mouth is 1.44 µg/mL. These values are higher than the MIC values against all *Candida* spp. and reflect the antifungal efficacy. In addition, the antibacterial effect of NMP and borneol in formulations against both *S. aureus* and *E. coli* [64,75] resulted in an efficient inhibitory effect on both bacteria and fungi.

## 3. Conclusions

Clear and less-viscous solutions were attained from preparation of clotrimazole-loaded ISGs comprising clove oil as a co-active agent and NMP as a solvent. These ISGs with acceptable pH values could be used for spraying in the oral cavity. Increasing the borneol content slightly diminished the density, surface tension, water tolerance, and spray angle, whereas it increased the viscosity and gel formation. Significantly higher contact angles on agarose gel and porcine buccal mucosa were promoted by NMP removal from these borneol-based ISGs, and thereafter, matrix formation retarded droplet spreading. Clotrimazole-loaded ISG containing 40% *w*/*w* borneol (40BZCN) enabled spraying and rapidly transformed to gel. The borneol matrix generated from this ISG extended the drug penetration through the synthetic membrane and porcine buccal membrane, indicating its potential antifungal activity against invading fungi. Clotrimazole-loaded ISG demonstrated an efficacious inhibition of growth against *S. aureus*, *E. coli*, *C. albicans*, *C. krusei*, *C. lusitaniae*, and *C. tropicalis* for oropharyngeal candidiasis treatment. Thus, this developed 40% *w*/*w* borneol-based ISG exhibited great potential as a promising clotrimazole-loaded spray for oropharyngeal candidiasis treatment.

## 4. Materials and Methods

### 4.1. Materials

Clotrimazole (batch no. 20001304, Lambrochem, Cologno Monzese, Italy) was used as an antifungal agent. Borneol from Chareorsuk-osop herbal shop, Nakhon Pathom, Thailand, was employed as a main matrix-forming agent. NMP (Lot No. 144560-118, QReC, Auckland, New Zealand) was used as the vehicle. Clove oil was procured from PC Drug, Bangkok, Thailand. Potassium dihydrogen orthophosphate (Lot No. E23W60) and sodium hydroxide (Lot No. AF310204) from Ajax Finechem, New South Wales, Australia, were dissolved in water for preparing PBS with pH 6.8. Agarose (Lot No. H7014714, Vivantis, Selangor Darul Ehsan, Malaysia) was acquired to prepare an agarose well. *S. aureus*, *E. coli*, *C. albicans*, *C. krusei*, *C. lusitaniae*, and *C. tropicalis* were obtained from the Ministry of Public Health, Mueang, Nonthaburi District, Thailand. Tryptic soy agar (TSA) and tryptic soy broth (TSB) (Difco, Detroit, MI, USA) were used as media for antibacterial testing. Sabouraud dextrose agar (SDA) and Sabouraud dextrose broth (SDB) (Difco, Detroit, MI, USA) were employed for the antifungal test.

### 4.2. Preparation of the ISGs

A simple mixing method was used to prepare the ISG solutions and their comparative groups. From the components of the formulations in Table 4, the different concentrations of borneol (20–50% *w*/*w*) were individually combined with 1% *w*/*w* clove oil and 1% *w*/*w* clotrimazole and subsequently dissolved in NMP by stirring gently and continuously for 3 h with a magnetic stirrer (Benchmark H3770-HS-E-Digital Hotplate Stirrer, Benchmark Scientific Inc., Sayreville, NJ, USA) at room temperature until clear solutions were obtained. Moreover, CN, ZN, ZCN, and 40BN were prepared using the same method and applied as the comparative groups. The detail of the components of the formulations is presented in Table 4.

### 4.3. Evaluations

#### 4.3.1. pH, Density, and Viscosities

The formulations were checked for their pH values using a pH meter (Seven Compact, Mettler Toledo, East Bunker Ct Vernon Hills, IL, USA) at room temperature in triplicate. The density measurement of all prepared formulations at room temperature was undertaken using a pycnometer (Densito 30PX, Mettler Toled Ltd., PortableLab™, East Bunker Ct Vernon Hills, IL, USA) (*n* = 3). Viscosity and shear stress were assessed using a viscometer (Brookfield Engineering Laboratories Inc., Middleborough, MA, USA) at 25 °C (*n* = 3).

#### 4.3.2. Surface Tension and Contact Angle

The determinations for contact angle values of the formulations on glass, agarose gel, and the bulge of the porcine surfaces were undertaken using a drop-shape analyzer (FTA 1000, First Ten Angstroms, Newark, CA, USA) with a pump-out rate of 1.9 µL/sec, and the obtained contact angle at 5 seconds was noted (*n* = 3). The finished cut tissue for fresh bulge of the porcine cheek was purchased from the slaughterhouse (Nakhon Pathom, Thailand). The buccal mucosa was attained after removal of subcutaneous fatty layer and connective tissues from the bulge of porcine cheek tissue. The freshly prepared mucosa was washed with PBS and wrapped in aluminum foil prior to use. The mucosa was used within 2 days of preparation. Moreover, surface tension was checked from a pendant drop of these liquid ISGs suspended in the air after injection using the drop-shape analyzer using the same pump-out rate performed in triplicate.

#### 4.3.3. Spray Pattern

The developed dosage form was evaluated both for spray pattern and spray angle because these ISGs should be administrated via spraying into oral cavity. The spray pattern of formulations was photographed after spraying through the spray nozzle into the air. All formulations were tested for their spray angles to indicate and compare their spray behaviors. The spray angle was measured at the position close to the spray nozzle. In addition, plain white paper was used as a screen 30 cm away from the nozzle to check the spray pattern of droplets spread on its surface. After spraying once, the spray distribution characteristics shown on this paper were compared between formulations.

#### 4.3.4. Water Tolerance Measurement

The durability of the formulation against water’s induction of phase separation was investigated from water titration. This investigation was performed by adding 2.5 g of the ISG into a test tube. The 20 µL deionized water was then dropped using a micropipette and mixed via a vortex mixer until it became turbid at 25 °C, and values were calculated using Equation (1) (*n* = 3).

(1) $$\%water\;tolerance\;=\;\frac{water\;amount(g)}{sample\;amount\;\left(g\right)\;+\;water\;amount\;(g)}\times 100\%$$

#### 4.3.5. Gel-Formation Study

The transformation from solution into gel via water induction was evaluated by injecting ISG through a 1 mL syringe with an 18-gauge needle into simulated saliva PBS (pH 6.8). The images indicating the gradual alteration from initial solution to gel or matrix-like form were recorded at different time intervals as a more turbid layer occurred with time after the aqueous phase diffused to induce the phase separation of the formula. This transformation was recorded at different time intervals (5, 10, 15, 20, and 25 min). Moreover, the microscopic aspect of above phase transition was conducted with 150 µL ISGs after exposure in the agarose well (diameter of 7 mm). The medium in agarose was simulated saliva PBS (pH 6.8); therefore, it represented the buccal area in human beings, which is covered with saliva. This change in the cross-sectional view of borneol gel in the agarose well was photographed under a stereo microscope (SZX10, Olympus Corp., Tokyo, Japan) at 1, 5, 10, 20, and 30 min with SZX10 Series software.4.3.5.

#### 4.3.6. In Vitro Drug Release, Permeation, and Drug Retention Studies

The release of clotrimazole through the nylon membrane and the permeation of clotrimazole through the porcine buccal mucosa from 40BZCN were tested and compared with ZN. A Franz-type diffusion cell (DHC-6T, Logan instrument corp., Somerset, NJ, USA.) was used for these investigations. The nylon membrane (Whatman™, M&P IMPEX Ltd., Bangkok, Thailand) with a pore size of 0.45 μm was used for the drug release test. In addition, the bulge of the porcine cheek from the general slaughterhouse (Nakhon Pathom, Thailand) was used as a membrane model simulating a human cheek bulge for the drug permeation experiment. The preparation of buccal mucosa from the bulge of the porcine cheek was performed with the method previously described in 4.3.2. Then, PBS pH 6.8 was added into the Franz receptor cells. The nylon or porcine buccal membrane was mounted in the horizontal glass of the Franz diffusion cells. Then, 0.25 g of formulation was added into the donor section. The system was set with a controlled temperature of 37 °C using water flow through the cells and with constant-speed stirring of the receptor solution to simulate the drug release behavior from the oral cavity. The 1 mL aliquots of the solution from the receptor cells were sampled, kept it a vial, and replaced in the receptor cells with 1 mL of fresh PBS with pH 6.8. The concentration of drug release was determined using high-performance liquid chromatography (HPLC) (Agilent 1220, Santa Clara, CA, USA.). The instrument was coupled with a UV detector and analyzed at 229 nm and C18 (150 × 4.6 mm) column. Methanol: water (90:10) was used as the mobile phase, with the flow rate and injection volume of 1.0 mL/min and 20 μL, respectively. The concentration of mean drug release through the nylon membrane ± S.D. was determined (*n* = 3). The lag time was checked, and the flux was calculated from the permeation test of ZN and 40BZCN through the porcine buccal membrane (*n* = 3). After the complete drug permeation experiment, the formulation matrix that occurred on the tissue was collected and dissolved in absolute ethanol for 1 h. The content of clotrimazole in this part was determined using the HPLC method using same condition as mentioned above for finding the remaining drug in the formulation matrix. Moreover, the tissue was cut into fine pieces. It was then sonicated and dissolved in absolute ethanol for 1 h before determining drug content with the aforementioned technique for finding the remaining drug in tissue. Eventually, the drug contents in three compartments of both formulations were compared.

#### 4.3.7. Antimicrobial Activity

All prepared ISG formulations were assessed for their antimicrobial activities against *S. aureus* ATCC 6538, *E. coli* ATCC 25922, *C. albicans* ATCC 17110, *C. krusei* TISTR 5259, *C. lusitaniae* TISTR 5156, and *C. tropicalis* TISTR 5306 using the agar cup diffusion method. Bacteria inocula were incubated for 36 h in TSB; meanwhile, the fungus was incubated in SDB for 72 h in TSB, during which the turbidity of the broth suspensions was calibrated using the 0.5 McFarland standard. Subsequently, the obtained broth suspensions of bacteria were swab spread on the TSA plates, whereas the inoculum of calibrated fungi was swab spread on SDA. Sterilized cylindrical cups were carefully placed on the swabbed agar before the 200 µL aliquot of prepared formulations was individually added to the cup and incubated at 37 °C. The diameter of the inhibition zone was measured after incubating the bacteria and fungi for 24 h and 72 h, respectively, using a ruler (*n* = 3).

### 4.4. Statistical Analysis

Statistical significance was assessed by one-way analysis of variance (ANOVA) followed by the Tukey test. The analysis was conducted using SPSS for Windows (version 11.5). The significance level was set at *p* < 0.05.

## Figures and Tables

**Table 1:** Physical properties of clotrimazole-loaded borneol-based ISG and related formulations. Results are shown as mean values ± SD, (*n* = 3).

Formulation	pH	Density (g/cm^3^)	Surface Tension (mN/m)	Viscosity (cPs)
NMP	11.07 ± 0.07	1.03 ± 0.01	37.36 ± 0.74	2.40 ± 0.06
CN	10.68 ± 0.04	1.03 ± 0.01	37.47 ± 0.79	2.54 ± 0.07
ZN	7.59 ± 0.01 ^a,b^	1.03 ± 0.00	38.34 ± 0.95	2.62 ± 0.10
ZCN	7.43 ± 0.02 ^a,b^	1.03 ± 0.00	37.77 ± 0.86	2.44 ± 0.03
20BZCN	6.61 ± 0.01 ^a,b^	1.02 ± 0.01	33.32 ± 0.93 ^c^	3.83 ± 0.02 ^d^
30BZCN	6.22 ± 0.01 ^a,b^	1.01 ± 0.01	32.39 ± 1.10 ^c^	4.31 ± 0.01 ^d^
40BZCN	5.89 ± 0.02 ^a,b^	1.01 ± 0.00	31.42 ± 0.92 ^c^	5.35 ± 0.03 ^d^
50BZCN	5.59 ± 0.01 ^a,b^	1.00 ± 0.01	30.19 ± 0.61 ^c^	7.91 ± 0.11 ^d^
40BN	5.91 ± 0.03 ^a,b^	1.01 ± 0.01	32.19 ± 0.70 ^c^	5.02 ± 0.05 ^d^

The superscripts ^a^, ^b^, ^c^, and ^d^ in the columns represent a significant difference (*p* < 0.05) compared with pH of NMP, pH of CN, surface tension of ZCN, and viscosity of ZCN, respectively.

**Table 2:** Clotrimazole remaining in different parts of Franz cell equipment of 40BZCN compared with ZN. Results are shown as % mean values ± SD, (*n* = 6).

Place of Drug for Analyzing	Clotrimazole Remains (%)	
	40BZCN	ZN
Borneol matrix over buccal porcine (Donor chamber)	77.10 ± 5.72 *	N/A
Inside buccal porcine (Membrane)	16.39 ± 5.65 *	75.51 ± 9.81 *
Under buccal porcine (Receptor chamber)	3.44 ± 1.56	21.56 ± 4.73

N/A represents not applicable. * represents a significant difference (*p* < 0.01) compared with receptor chamber of each formulation.

**Table 3:** Inhibition zone diameter of clotrimazole-loaded borneol-based ISG and related formulations against *S. aureus*, *E. coli*, *C. albicans*, *C. krusei*, *C. lusitaniae*, and *C.* tropicalis (*n* = 3).

Microbes	Inhibition Zone Diameter (Mean ± SD) (mm)							
	NMP	CN	ZN	ZCN	20BZCN	30BZCN	40BZCN	50BZCN
*S. aureus* ATCC 6538	10.67 ± 1.53	14.17 ± 1.04	21.33 ± 1.53	20.00 ± 1.00	20.33 ± 0.58	17.00 ± 1.00	16.00 ± 1.00	13.83 ± 1.26
*E. coli* ATCC 25922	17.67 ± 1.53	19.00 ± 1.00	18.00 ± 1.00	17.00 ± 1.00	14.00 ± 1.00	12.33 ± 1.53	11.00 ± 1.00	8.17 ± 0.29
*C. albicans* ATCC 17110	28.33 ± 1.53	30.33 ± 0.58	39.33 ± 0.58	39.00 ± 1.00	43.67 ± 1.15	42.67 ± 0.58 ^a^	42.33 ± 0.58 ^a^	42.67 ± 0.58 ^a^
*C. krusei* TISTR 5259	14.33 ± 1.53	14.00 ± 2.00	37.00 ± 2.00 ^b^	32.00 ± 1.00	38.67 ± 0.58	36.33 ± 1.53 ^b^	34.67 ± 1.15	31.00 ± 1.73
*C. lusitaniae* TISTR 5156	30.00 ± 1.00	30.67 ± 0.58	38.33 ± 1.15 ^c^	38.33 ± 0.58 ^c^	39.33 ± 1.53	37.00 ± 1.00 ^c^	35.33 ± 0.58	33.33 ± 0.58
*C. tropicalis* TISTR 5306	27.67 ± 0.58	29.33 ± 0.58	36.33 ± 0.58	37.00 ± 1.00	40.67 ± 1.15	39.33 ± 0.58 ^d^	36.33 ± 0.58	35.00 ± 1.00

The superscripts ^a^, ^b^, ^c^, and ^d^ represent a significant difference (*p* < 0.05) compared with 20BZCN.

**Table 4:** Compositions of clotrimazole-loaded borneol-based ISG and drug-free formulations.

Formulation	Amount (%*w*/*w*)			
	Clotrimazole	Clove oil	Borneol	NMP
NMP	-	-	-	100
CN	-	1	-	99
ZN	1	-	-	99
ZCN	1	1	-	98
20BZCN	1	1	20	78
30BZCN	1	1	30	68
40BZCN	1	1	40	58
50BZCN	1	1	50	48
40BN	-	-	40	60

## Data Availability

The data presented in this study are available on request from the corresponding author.
